# Radical Scavenging Activity of Olive Oil Phenolic Antioxidants in Oil or Water Phase during the Oxidation of O/W Emulsions: An Oxidomics Approach

**DOI:** 10.3390/antiox9100996

**Published:** 2020-10-15

**Authors:** Vito Michele Paradiso, Federica Flamminii, Paola Pittia, Francesco Caponio, Carla Di Mattia

**Affiliations:** 1 Department of Biological and Environmental Sciences and Technologies, University of Salento, I-73100 Lecce, Italy; 2 Department of Soil, Plant and Food Sciences, University of Bari, I-70126 Bari, Italy; francesco.caponio@uniba.it; 3 Faculty of Bioscience and Technology for Agriculture, Food and Environment, University of Teramo, I-64100 Teramo, Italy; fflamminii@unite.it (F.F.); ppittia@unite.it (P.P.)

**Keywords:** O/W emulsions, olive oil phenolic antioxidants, oxidative stability, oxidomics, hydroperoxides, volatile compounds

## Abstract

Omics approaches are recently being applied also in food lipid oxidation, to increase knowledge of oxidation and antioxidation mechanisms. The so-called oxidomics throws a wider spot of light on the complex patterns of reactions taking place in food lipids, especially in dispersed systems. This research aimed to investigate the radical scavenging activity of olive oil phenolic antioxidants (OPAs) in O/W emulsions, as affected by the phase in which they were added. This allowed one to assess whether different behaviors could be expected from antioxidants originally present in phenolic-rich olive oils compared to natural antioxidants added in the water phase during emulsion production. Hydroperoxide decomposition kinetics and the analysis of volatile pattern provided an outline of antioxidation mechanisms. Though being effective in slowing down oxidation when added both in the oil and water phase, OPAs interfered in different ways with oxidation pathways, based on the phase in which they were added. OPAs added to the water phase were more effective in slowing down hydroperoxide decomposition due to the hydrophilic radical initiator. On the other hand, OPAs present in the oil were more effective in preventing radical propagation, with relevant consequences on the volatile pattern.

## 1. Introduction

In formulated food products, lipids are often present in the form of a dispersion, being the oil-in-water emulsions the most common structures. From the manufacture to their end-use, food emulsions are exposed to a broad range of physico-chemical treatments, most of them in the presence of oxygen, with the consequence that chemically reactive components may be oxidized [1]. Radicals can thus be present as consequences either of biochemical pathways or generated by processing/formulation and, once formed, are responsible for the propagation of the oxidative reactions. Lipid oxidation involves indeed a free radical chain reaction whose final products have a deleterious effect on the technological, sensory, and nutritional qualities of foods. Among the strategies that can be undertaken by the industries to delay oxidation phenomena, the addition of antioxidant compounds is one of the most common; however, antioxidants polarity, partition behavior, location within the emulsion, and reactivity substantially affect their effectiveness and functionality in a multiphasic system, due to the essentially interfacial nature of oxidation in food dispersions. In the attempt to explain the activity of antioxidants in emulsions several theories have been developed, starting from the Polar Paradox, by Porter in 1980, and its more recent revisited version [2,3], to the “cut-off” effect or the “pseudo-phase” model. Such theories, despite differing in either the approach or the proposed mechanism, emphasize the role of the interfacial location of the antioxidant compound but at the same time point out that its behavior in emulsion is still far from being understood. Indeed, a gap between the potential of a certain antioxidant molecule and its efficacy in a complex multiphasic system still exists.

Recently, Decker et al. [4] pointed out various hurdles in predicting the effectiveness of antioxidants in real emulsions: the involvement in non-free radical scavenging reactions, the presence of different types of interfaces, inherent obstacles in studying lipid droplets properties, and varying oxidation kinetics in different droplets. Moreover, several contributions have recently suggested the expansion of traditional analytical patterns to a wider range of oxidation products to better understand oxidative phenomena [5,6,7]. Three distinct viewpoints by Paradiso, Villeneuve, and Laguerre discussed the need for a new step to study lipid oxidation in emulsified systems by focusing on different aspects [8,9,10]. They stressed the importance of investigating some crucial parameters, up to now underestimated, that may govern antioxidant efficiency like the role of the transport of reactive species by surfactant micelles, the impact of smaller droplets in poly-dispersed systems, the application of multiparametric approaches to the analysis of oxidation reactions, and the development of spatially and temporally resolved models for lipid oxidation. Additionally, Paradiso suggested also the need for epistemological and experimental approaches by applying an interdisciplinary perspective. In particular, oxidomics, being an inductive, hypothesis-generating, circular approach involving multiparametric analyses, can provide the means to deepen the understanding of lipid oxidation and antioxidation processes in complex food matrices [11]. Gómez-Cortés and Camiña successfully applied this approach to differentiate oxidation in oils of different origin [12].

This work was, thus, aimed to apply an omics approach to investigate the radical scavenging activity of olive oil phenolic antioxidants added either in the oil or water phase during the oxidation of O/W emulsions. Besides allowing to better understand the antioxidant mechanisms in dispersed systems, this research was also intended to assess whether different behaviors could be expected from antioxidants originally present in phenolic-rich olive oils compared to natural antioxidants added in the water phase during emulsion production.

## 2. Materials and Methods

### 2.1. Materials

Lyophilized β-lactoglobulin (BLG) from bovine milk, with a purity of 97.2%, was kindly donated by the Technische Universitat Munchen (TUM) (München, Germany) (lot. n. CR 47) and obtained following an optimized isolation procedure based on thermal precipitation and separation by microfiltration and ultrafiltration, as reported by Toro-Sierra and others [13]. The water phase consisted of using double distilled and sterilized water. The oil phase of emulsions consisted of a highly purified olive oil (Sigma-Aldrich Ltd., St. Luis, MO, USA) from a single batch, stored in sealed bottles in the dark at 15 °C. The olive oil phenolic antioxidants (OPA) were supplied by the Department of Agricultural and Food Sciences of the University of Bologna, (Bologna, Italy); the phenolic extract was obtained by liquid–liquid extraction with pure water from a high phenolic extra virgin olive oil (cv. Coratina), followed by freeze-drying and re-solubilization in water to get a final concentration of 1700 mg/L, as evaluated according to the International Olive Council method [14]. The phenolic composition of the extract was evaluated by High-Performance Liquid Chromatography with Diode-Array Detector (HPLC-DAD) by using the analytical procedure described in the work by Reboredo-Rodriguez and others [15] and was as follows: hydroxytyrosol (≈17%), tyrosol (≈23%), oleuropein aglycone (≈20%), decarboxymethyloleuropein aglycon (≈15%), and decarboxymethylligstroside aglycon (≈25%). The same analytical method proved that the highly purified olive oil did not contain antioxidants. All the reagents were of analytical grade. 

### 2.2. Emulsions Preparation and Characterization

Olive oil-in-water (O/W) emulsions (20%, *w*/*w*) were obtained in two steps: pre-emulsification with a high shear mixing device (Ultra-Turrax yellow line DI25 basic, Ika-Werke GmbH & Co, Staufen, Germany) at 13,500 rpm for 1 min was followed by high-pressure homogenization using a Panda Plus 2000 homogenizer (GEA Niro Soavi, Parma, Italy) by applying five homogenization cycles at 150 bar. The aqueous phase was a 0.5% (*w*/*w*) BLG solution in 50 mM phosphate buffer (pH 7.0), added with 0.03% (*w*/*w*) sodium azide (NaN_3_) prior to emulsification as antibacterial agent.

Model O/W emulsified systems were prepared by mixing OPAs either in the continuous phase (W) or in the dispersed phase (O) prior to homogenization; considering the phase volume ratio (ϕ = 0.2), the amount added in each phase differed in order to get a final OPA concentration of 100 mg L^−1^ on the emulsion volume basis. 

For the evaluation of the total phenolic content and the antiradical activity, the emulsions were submitted to two cycles of freezing, thawing, and centrifugation at 13,000× *g* for 5 min to break the emulsions and separate the continuous aqueous phase.

The particle size and droplets surface area of the emulsions were determined by laser diffraction analysis through the Mastersizer Hydro 3000 (Malvern Instruments Ltd., Worcestershire, UK). An amount of emulsion was added to the dispersion unit (Hydro 2000S, Malvern Instruments Ltd., Worcestershire, UK) containing distilled water until an obscuration of 5–6% was reached. Each sample was analyzed in triplicate and five records for each measurement were collected. A refractive index of 1.59 and an absorption of 0.01 were defined for the optical properties of the sample. Droplet size measurements were reported as specific surface area (SSA) and volume mean diameter (D_4,3_).

### 2.3. Oxidative Stability of O/W Emulsions

The RapidOxy equipment (Anton Paar, Blankenfelde-Mahlow, Germany) was used to assess the oxidative stability of emulsions. Samples were submitted to forced oxidation (T = 140 °C, P_O2_ = 700 kPa) and their induction time was measured as the time needed for a 10% drop of the oxygen pressure.

### 2.4. Accelerated Oxidation of O/W Emulsions

Accelerated oxidation was induced in emulsions by using the hydrophilic radical initiator 2,2′-Azobis(2-methylpropionamidine) dihydrochloride (AAPH). A 1 mM solution of the initiator was prepared in ultra-pure water and added to the emulsions. Aliquots (1 mL) of emulsions were distributed in headspace vials (12 mL) sealed with an aluminum cap and butyl rubber septum. Three individual vials were sampled at 0, 2, 4, 7, 14, 28, and 57 days and submitted to the analyses of lipid hydroperoxides. Three additional vials were used for Headspace Solid-Phase Microextraction-Gas Chromatography/Mass Spectrometry (HS-SPME-GC/MS) analysis after 0, 2, 4, 7, and 14 days.

### 2.5. Total Phenolic Content

The total phenolic content of the continuous phase of O/W emulsions was determined spectrophotometrically using the Folin–Ciocalteu method adapted from [16]. The sample (100 µL) was added to distilled water (4800 µL) and Folin–Ciocalteu reagent (500 µL). After 3 min, 1500 µL of sodium carbonate solution 25% (*w*/*v*) was added and filled with distilled water up to 10 mL. Absorption at 765 nm was measured after resting for 1 h in the dark by a UV-vis spectrophotometer (Lambda Bio 20, Perkin–Elmer, Waltham, MA, USA). Gallic acid was used for calibration. 

### 2.6. Antiradical Activity

The antiradical activity of the aqueous phase was measured by a radical cation decolorization assay as reported by Re et al. [17] with some modifications. Fifteen microliters of properly diluted aqueous phase were mixed with 1.5 mL of 2,2′-azinobis-(3-ethylbenzothiazoline-6-sulfonic acid) (ABTS^+•^) radical. The antiradical activity was measured as the bleaching of the sample–radical mix, measuring the absorbance at 734 nm wavelength with a spectrophotometer (Lambda Bio 20, Perkin-Elmer, Waltham, MA, USA). Results were expressed as μmol TE mL^−1^ of the continuous phase, using Trolox for calibration.

### 2.7. Lipid Hydroperoxides

Lipid hydroperoxides were measured according to the method proposed by Shantha and Decker [18]. An aliquot of the emulsion (0.3 mL) was mixed with 1.5 mL of organic solvent (isooctane/isopropanol, 2:1, *v*/*v*), vortexed three times for 30 s, and centrifuged for 2 min at 1000 rpm. The supernatant was taken (200 µL) and added to 2.8 mL of methanol:1-butanol (3:1, *v*/*v*), 15 µL of 3.94 M ammonium thiocyanate, and 15 µL of ferrous iron solution (prepared by adding equal amounts of 0.132 M BaCl_2_ and 0.144 M FeSO_4_). Sample absorbance at 510 nm was measured after 20 min, with a spectrophotometer (Cary 60 UV-Vis, Agilent Technologies, Santa Clara, CA, USA). A standard calibration curve prepared with hydrogen peroxide was used for hydroperoxide quantitation.

### 2.8. FTIR Analysis

Spectra of all emulsions were acquired using a Nicolet iS50 FTIR (Thermo Fisher Scientific, Waltham, MA, USA) spectrometer equipped with a Smart iTR attenuated total reflectance (ATR) sampling accessory. Transmittance was measured against air as a background over the wavenumber range of 4000 to 650 cm^−1^ (4 cm^−1^ resolution). Spectra were obtained by co-adding and averaging 64 scans. Approximately 100 µL of emulsion were uniformly spread over the ZnSe crystal surface for spectra recording. A cellulose tissue soaked in ethanol was used to clean the ATR crystal after each spectrum. The analysis was done for each sample in parallel with oxidation analyses. Smoothing using the Savitzky-Golay algorithm (15 points window) and subsequent standard normal variate normalization was carried out before plotting and comparing the spectra.

### 2.9. Volatile Compounds Analysis

Volatile compounds as markers of secondary oxidation reactions were extracted and analyzed by HS-SPME-GC/MS. A 75 µm carboxen/polydimethylsiloxane (CAR/PDMS) fiber (Supelco, PA, USA) was used for volatile compounds extraction by exposure in the headspace of the sample at 35 °C for 15 min. The fiber was then inserted into the injector port (set at 220 °C) for 2 min in splitless mode. The GC/MS analysis was carried out with an Agilent 6850 gas chromatograph coupled to an Agilent 5975 mass spectrometer (Agilent Technologies, Santa Clara, CA, USA). Analytes separation was obtained on an HP-Innowax capillary column (20 m × 0.18 mm, 0.18 µm film thickness, Agilent Technologies, Santa Clara, CA, USA) using helium as a gas carrier. The chromatographic conditions and detector operation conditions are reported in our previous paper [19]. Semiquantitative data were reported as peak areas.

### 2.10. Statistical Analysis

Analysis of variance (ANOVA), post-hoc Tukey’s HSD test (honestly significant difference), and non-linear regression fitting were performed using OriginPro 2020 (OriginLab, Northampton, MA, USA). 

## 3. Results

### 3.1. Oxidative Stability

The levels of OPA enrichment corresponded to those that could be provided to emulsions by the use of a high-quality extra virgin olive oil [20,21] containing roughly 450 mg kg^−1^ of oil, calculated considering the enrichment (100 mg L^−1^ of emulsion, the volume fraction (ϕ = 0.2), and average olive oil density (0.91 g cm^−3^).

**Figure 1:**
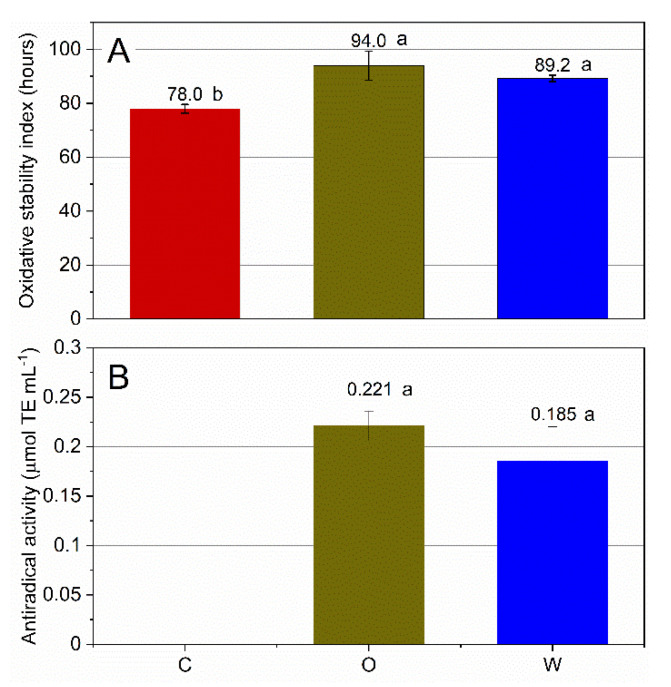
Oxidative stability (**A**) and antiradical activity (**B**) of O/W emulsions added with OPA. C, control emulsion; O, emulsion with OPA added to the oil phase; W, emulsion with OPA added to the water phase. Mean values ± standard deviation (*n* = 3). A Tukey’s HSD test for multiple comparisons was carried out, different small letters (a,b) mean a significant difference at *p* < 0.05.

The oxidative stability of the emulsions, characterized by similar droplet size and surface area (data not shown), was determined using an accelerated test under stressing temperature (120 °C) and oxygen pressure (6 atm) conditions. The OPAs, allowed to extend by 20.6% and 14.5% the oxidative stability of the emulsions, when added to the oil or water phase, respectively (Figure 1). The increase was significant (*p* < 0.01) in both cases, but no significant difference (*p* = 0.129) was observed between the two systems with antioxidants.

Such results can be considered in agreement with the ones of total phenolic content and antiradical activity, determined on the continuous phase of the emulsions after breakage of the systems. Indeed, whatever the phase of enrichment, the antiradical activity, determined by means of the ABTS radical cationic assay, revealed no significant differences between the O and W systems (0.221 ± 0.015 and 0.185 ± 0.035 µmol TE mL^−1^ when the extract was added in the dispersed and continuous phases, respectively). The same can be affirmed for the content of phenolic compounds, mainly made of secoiridoids, which partitioned in the aqueous phase to a comparable though significantly different (*p* < 0.01) extent (28% and 34% in the O and W emulsions, respectively).

Therefore, on the basis of the specific conditions of the accelerated test (i.e., high oxygen pressure and temperature) and of the antiradical activity assay, comparable oxygen consumption and antiradical activity could be inferred. This is not sufficient to conclude that OPA produced the same effects, irrespective of the phase in which they were added. In fact, besides chemical reactivity, partitioning also plays a key role in antioxidant real effectiveness [4]. Possible differences in oxidation patterns (oxidome) and their evolution during oxidation could not be evaluated and require a multiparametric, omics-based approach.

### 3.2. Hydroperoxides

**Figure 2:**
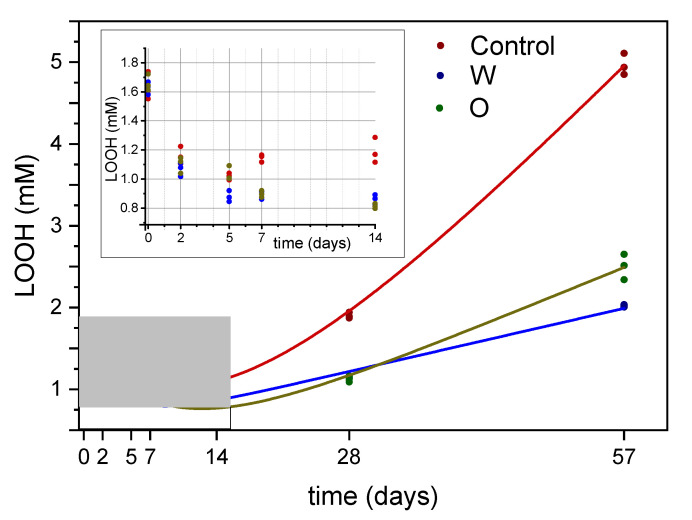
Trend of hydroperoxides in O/W emulsions, added with OPA, during accelerated oxidation test with AAPH radical initiator. Control, control emulsion; O, emulsion with OPA added to the oil phase; W, emulsion with OPA added to the water phase. Shah fitting is reported (see text for details on the model).

Figure 2 reports the trends of hydroperoxides in the emulsions submitted to radical-catalyzed oxidation. Though purified olive oil, free from oxidation products, was used for emulsion production, fresh emulsions contained about 1.6 mM hydroperoxides. This confirms the pro-oxidative effect of the emulsification process, which induces the oxidation of unsaturated fatty acids and the release of volatiles [22,23,24,25]. In the first stage of the oxidation test, hydroperoxides underwent a decrease, while the second phase of oxidation was characterized by an increase of hydroperoxides. The minimum fell for all three systems between 7 and 14 days.

A nonlinear curve fitting of the data was performed. A Shah model [26] was successfully applied to the datasets. This nonlinear regression model combines an exponential decay function with a linear function, according to the following equation:

$$y=a+bx+cr^{x}$$

where *x* represents the time, y the content of hydroperoxides and *a*, *b*, *c* and *r* are the parameters of the model. Table 1 reports the parameters and the performances of the three models. The values of *x*_0_ and *y*_0_ are the coordinates of the point of minimum in the curves and are calculated according to the following equations:

$$x_{0}=\frac{\ln(-b/(c\times \ln r))}{\ln r};\;y_{0}=a+bx_{0}+cr^{x_{0}}$$

An exponential decay function combined with a linear function resulted, therefore, effective as a phenomenological model for the hydroperoxides trend in the tested systems. Aragao et al. [27] used phenomenological models for the rise and fall of peroxide value during lipid oxidation. They observed that a decay factor superimposed to an accumulation term could provide appropriate models for oxidation data found in the literature. They also reported that the structure of the models and the magnitude of their terms depends on the specific system under evaluation, while the shape of the curve depends on the time scale of the experiment and on the different kinetics of degradation and accumulation. 

The importance of time scale in oxidation experiments has been pointed out in another study [11]. The behavior of hydroperoxides during radical-catalyzed oxidation of emulsions showed, therefore, two competing chemical processes: hydroperoxides exponential decay and their synthesis, each one prevailing on different time scales. The *b* term of the models (i.e., the linear regression coefficient) was higher in the control emulsion compared to those with added antioxidants. Emulsions with antioxidants in the water phase showed the lowest value for the *b* coefficient. Therefore, hydroperoxide synthesis was slower in these systems. This could be attributed to the hydrophilic nature of the pro-oxidant radicals: OPAs in the water phase were more effective in inactivating the AAPH-generated radicals. Moreover, the rate of hydroperoxide decay, expressed by the coefficient *c* and the base *r*, was higher in W emulsions compared to O emulsions, while C systems showed the lowest rates. On the other hand, C emulsions showed a higher value of hydroperoxides at the point of minimum (*y*_0_) compared to W and O emulsions. This could mean an overlay on hydroperoxide formation with existing hydroperoxides decomposition, determining lower rates of exponential decay. In O emulsions, hydroperoxide decomposition was effectively slowed down by antioxidants added in the oil phase, therefore neighboring hydroperoxides. As regards the x value of the point of minimum (*x*_0_), it was first reached by W emulsions (8.4 days), while in O emulsions it was calculated at 12.4 days. C emulsions showed an intermediate value (9.7 days), probably due to the concurring hydroperoxide generation, pointed out by the high *y*_0_ value, as stated above.

### 3.3. FTIR Oxidation Features

**Figure 3:**
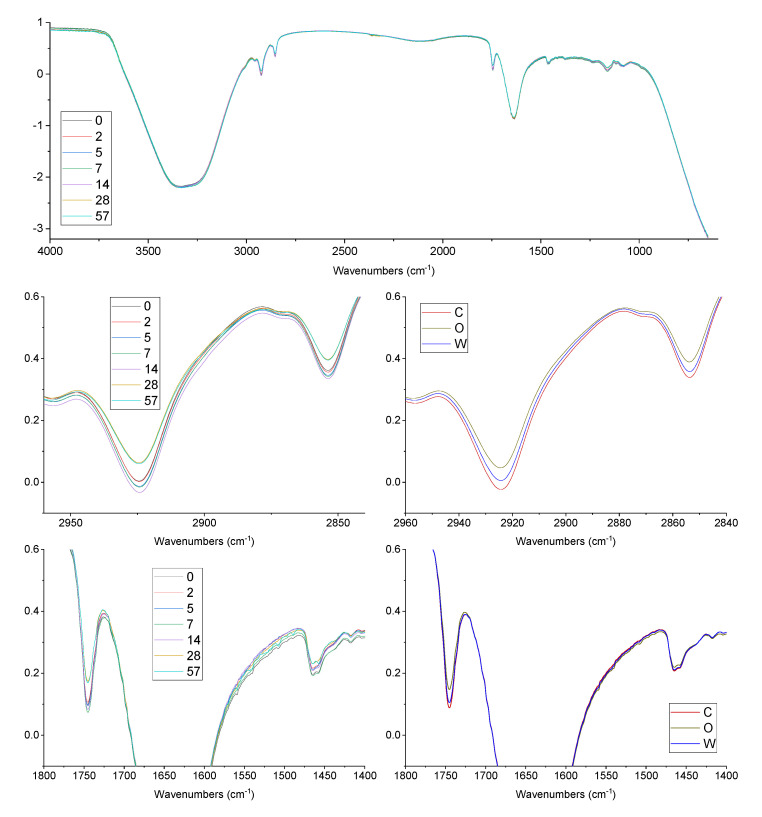
Trend of hydroperoxides in O/W emulsions, added with OPA, during accelerated oxidation test with AAPH radical initiator. C, control emulsion; O, emulsion with OPA added to the oil phase; W, emulsion with OPA added to the water phase. Shah fitting is reported (see text for details on the model).

Figure 3 reports the mean FTIR spectra of the emulsions during oxidation, averaged per either days of oxidation or emulsion system. Changes in the bands at 2925 and 2852 cm^−1^ were observed (related to asymmetric and symmetric stretching vibrations of methylene, −CH_2_, and methyl, −CH_3_), attributable to time and antioxidants addition. Transmittance decreased in the first 14 days, while after 28 and 57 days it was higher than at the beginning of oxidation. O emulsions showed the highest values of transmittance, while the lowest values were observed in the C emulsions. The decrease of transmittance in these bands as oxidation occurs was reported by other authors [28,29]. The J-shaped trend of the transmittance during oxidation observed in this study could be attributed to the wide time scale of the oxidation experiment. Though no statistical correlation was observed, an analogy with hydroperoxides can be observed. A similar trend during oxidation was observed for the band at 1747 cm^−1^, attributed to the stretching vibrations of the carbonyl group of triglyceride esters (−C=O), which is attended to increase during oxidation [29,30]. Again, O emulsions showed the highest values of transmittance, while the lowest values were observed in C emulsions.

A recent paper by Daoud et al. [30] used FTIR spectral features of O/W emulsions to monitor oxidation for 15 days, with both radical (AAPH) and iron initiation. Authors used tuna oil, rich in highly polyunsaturated fatty acids, so conjugated dienes formation was effectively monitored through FTIR analysis. The full spectrum and some spectral regions (1800 to 1550 cm^−1^ and 1500 to 900 cm^−1^) were related to the appearance of conjugated dienes, typical of polyunsaturated fatty acids oxidation. It is interesting to compare the findings: the trends observed in the present study were similar for the band at 1747 cm^−1^ (from 0 to 14 days), while opposite tendencies were observed for the bands at 2925 cm^−1^ and 2852 cm^−1^. These differences could be related to the different oils used (either highly polyunsaturated or rich in monounsaturated fatty acids) and to the time frame considered since a certain inversion of trends was observed in the present study after 14 days of oxidation.

### 3.4. Focus on the Hydroperoxide Decay Phase

**Figure 4:**
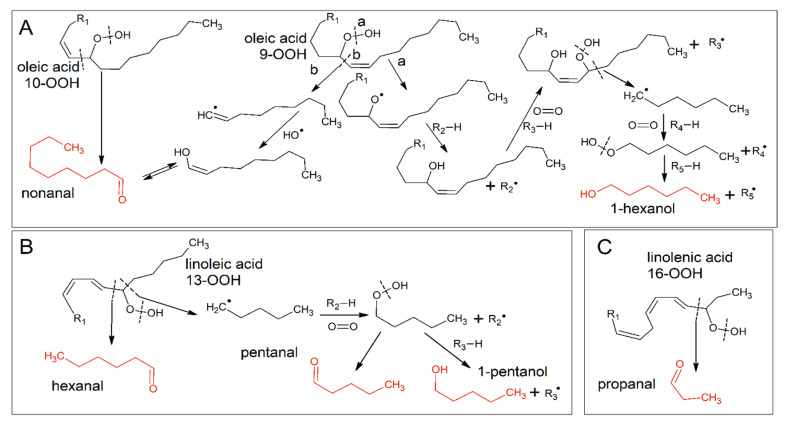
Formation pathways of the volatile compounds (in red) monitored during accelerated oxidation of O/W emulsions. (**A**) Oleic acid-derived compounds; (**B**) linoleic acid-derived compounds; (**C**) linolenic acid-derived compounds.

Data related to headspace volatile compounds were collected during the hydroperoxide decay phase (ca. 0 to 14 days), in order to gain further information about the antiradical mechanisms of the OPA. To this aim, five volatile markers were chosen, deriving from the decomposition of hydroperoxides from oleic (Figure 4A), linoleic (Figure 4B), and linolenic (Figure 4C) acids [31,32]. The choice of more than one volatile marker (usually hexanal) would allow for the obtaining of a more complete picture of the occurring phenomena [8,19,33].

**Figure 5:**
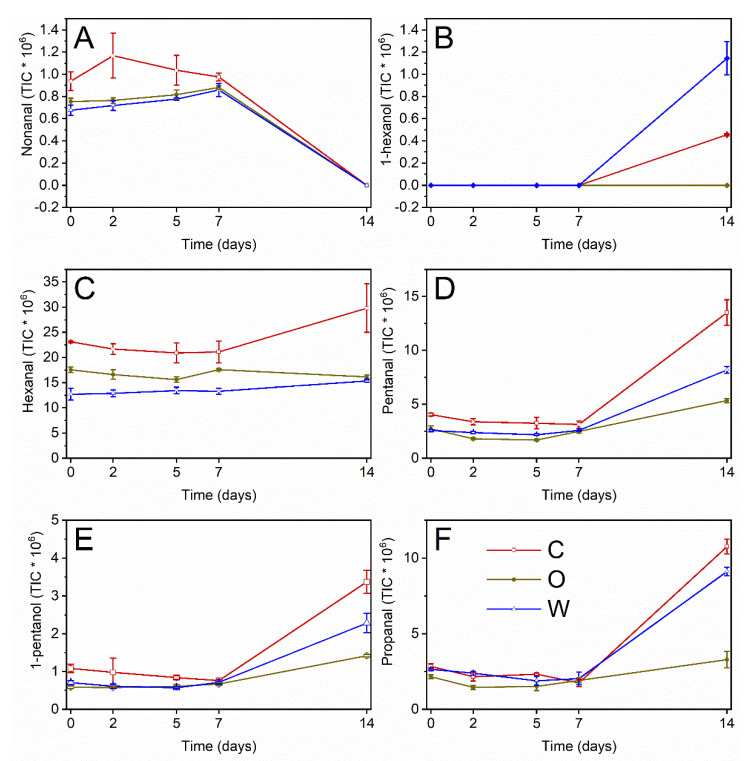
Trends of selected volatile compounds during accelerated oxidation of O/W emulsions added with OPAs. Mean values ± standard deviation (*n* = 3). C, control emulsion; O, emulsion with OPA added to the oil phase; W, emulsion with OPA added to the water phase. (**A**) nonanal; (**B**) 1-hexanol; (**C**) hexanal; (**D**) pentanal; (**E**) 1-pentanol; (**F**) propanal.

Figure 5 shows the trends of the selected volatile oxidation markers, while Table 2 reports the results of the multiple comparisons by Tukey’s HSD test. Nonanal and 1-hexanol are decomposition products of the monounsaturated oleic acid, by far the most abundant fatty acid in olive oils (Figure 5A). Nevertheless, their formation pathways are quite different. Nonanal is the most abundant decomposition product of oleic acid [31,32]; this aldehyde can derive from both 9- and 10-hydroperoxides. In the latter case, it is the results of the direct breakdown of the hydroperoxide, while its formation from 9-OOH requires a radical intermediate. Nonanal showed an unexpected trend. Freshly obtained emulsions showed significant differences in nonanal levels, pointing out a possible protective effect of the OPA towards oxidation induced by the process. In the early oxidation process, nonanal underwent an increase in control emulsions after two days of radical initiated oxidation, while it linearly increased (*p* < 0.05) in the first week in emulsions with OPA (slopes of the increase were not different, data not shown), though keeping at lower levels than in C emulsions. These trends corresponded to the rapid hydroperoxide decline, showing that oleic acid hydroperoxides were involved in this early stage. After 14 days of oxidation, nonanal disappeared from the headspace of the samples. Proteins in emulsions, and specifically BLG, have been reported to interact with aldehydes. Covalent interactions have been demonstrated to involve a,b-monounsaturated aldehydes [22,34]. Covalent binding of saturated aldehydes is controversial and was reported in aqueous systems and high aldehyde concentrations [22,34]. However, interactions of compounds with BLG in emulsions have been reported and attributed to the hydrophobic cavity [35], particularly for elongated structures [36]. The interactions were mainly related to chain length and compound hydrophobicity [37,38]. Another oleic acid-derived compound is 1-hexanol, a secondary scission product whose formation pathway requires some radical intermediates [31,32]. This compound was not detected in the initial stage of hydroperoxide cleavage. It was detected only after 14 days of oxidation in W emulsions and, in lower amounts, in C samples, while in O emulsions it did not reach detectable levels. The effectiveness of OPA changed, therefore, they exerted a focused activity towards radical-mediated secondary reactions when added in the oil phase.

Being, by far, the most abundant polyunsaturated fatty acid in olive oils, linoleic acid is the main substrate for early oxidation. Among its oxidation products, hexanal was, as expected, the most relevant (Figure 5C). No clear increase was observed during 14 days of oxidation, except for the final levels in C emulsions. This could be attributed to certain binding to BLG though to a lesser extent than for the longer chain of nonanal overlapping with its formation from direct cleavage of 13-OOH of linoleic acid (Figure 4B). On the other hand, significant differences were observed among the different emulsified systems: OPAs decreased the levels of hexanal, mainly when added in the water phase. Hydroperoxides have well-known surface activity [10,39]: antioxidants in the water phase could be more effective in slowing down both hydroperoxide decomposition and hydroperoxide-dependent initiation of oxidation [10,40]. Different results were observed for pentanal (Figure 5D) and 1-pentanol (Figure 5E): these compounds increased at 14 days after a lag phase and showed the lowest increase in O emulsions. Again, these five-carbon compounds are secondary scission products, requiring a radical intermediate: OPAs in the oil phase resulted as more effective in inhibiting such kinds of reaction. It is noteworthy that considering the only hexanal, as usually appears in the literature, as a secondary oxidation marker, would have led to the misleading conclusion that OPAs in the water phase were more effective towards secondary oxidation. The adopted oxidomics approach, instead, allowed to depict a more complete view of the mechanisms of action of these molecules [5,8,11,12].

As an oxidation product of linolenic acid, propanal was considered. It is the product of direct cleavage of 16-OOH. It showed again a lag phase and an increase after 14 days, probably deriving from newly formed hydroperoxides. W emulsions reached levels lower than C emulsions, but the levels in O emulsion were by far the lowest. It should be considered that it reaches 0.7% of total fatty acids in the used purified olive oil (according to the manufacturer’s certificate of analysis). Therefore, linoleic acid hydroperoxides formation would more probably occur during the radical propagation in the oil droplet rather than during the attack by the radical initiator in the water phase, for mere probabilistic reasons.

**Figure 6:**
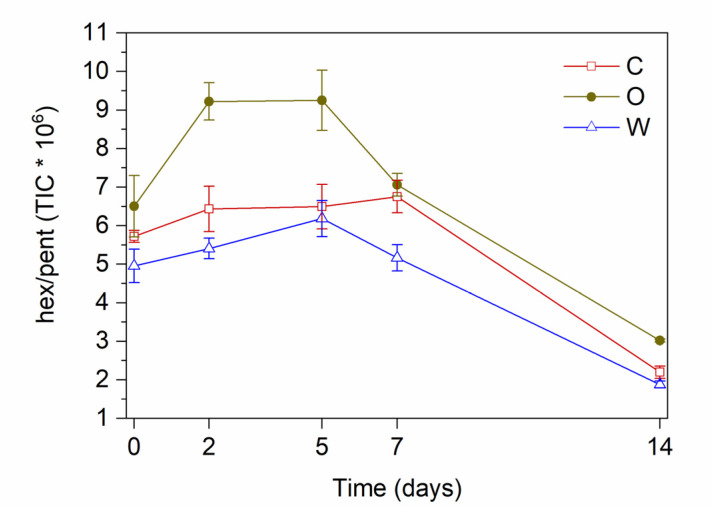
Hexanal/pentanal ratio during accelerated oxidation of O/W emulsions added with OPAs. Mean values ± standard deviation (*n* = 3). C, control emulsion; O, emulsion with OPAs added to the oil phase; W, emulsion with OPAs added to the water phase.

To better highlight the differences of behavior of the OPAs in water or in the oil phase, the ratio hexanal/pentanal (H/P) was plotted versus time for the tested emulsions (Figure 6). These compounds represent the main products of the secondary oxidation of linoleic acid from either direct hydroperoxide cleavage or radical-mediated degradation, respectively. The H/P ratio increased in the early stage of oxidation, in all cases, and subsequently decreased, probably due both to differential binding to BLG and different formation kinetics. Compared to C emulsions, W emulsions showed lower H/P ratio values, due to the inhibiting activity of OPA in the water phase towards the hydrophilic initiator; O emulsions showed, instead, higher values of H/P ratio with respect to the control, due to the inhibiting activity towards radical-mediated propagation. Carrasco-Pancorbo et al. [41] evaluated the antiradical activity of individual phenolic compounds in virgin olive oils. They observed that hydroxytyrosol showed the highest antiradical power compared to the other antioxidants. Several other researchers provide evidence that the catechol structure (characterizing hydroxytyrosol, oleuropein, and decarboxymethyloleuropein) exerts a marked inhibiting activity towards oxidation in emulsions [19,33,42]. In almost all cases, hydroxytyrosol confirmed in emulsions its outstanding antioxidant activity, though environmental factors (e.g., metals, pH) could determine relevant changes in the effects of olive oil phenolic antioxidants in emulsions [43].

These results point out that, though being effective in both cases towards radical oxidation processes, OPAs lead to different outcomes of oxidation when added to either oil or water. Such differing behaviors could be considered representative of olive oil-in-water emulsions made with either high quality extra virgin olive oils or with low-quality oils together with water-soluble natural antioxidant extracts [44].

## 4. Conclusions

An outline of antioxidation mechanisms in O/W emulsions added with OPAs either in the oil or in the water phase was obtained by means of an oxidomics approach. Though being effective in slowing down oxidation when added both in the oil and water phase, OPAs interfered in different ways with oxidation pathways, based on the phase in which they were added. OPAs added to the water phase were more effective in slowing down hydroperoxide decomposition by contrasting the activity of the hydrophilic radical initiator. On the other hand, OPAs added to the oil were more effective in preventing radical propagation, determining relevant differences in the volatile pattern. These results can be considered representative of the different outcomes of obtaining emulsions using either phenolic-rich oils or low-quality oils together with water-soluble phenolic extracts as natural antioxidant additives. This seems to offer a relevant alternative for food producers and has several implications. In fact, the use of high-quality extra virgin olive oils in food emulsions manufacturing is limited by the high cost of the ingredient and can be justified in high price products. On the other hand, the addition of hydrophilic antioxidants extracts could allow the use of oils with lower quality and lower price, providing increased stability to the final product. The extraction of antioxidants from olive oil by-products (e.g., wastewater, olive pomace, and leaves) and their consequent exploitation in view of a green economy paradigm could be another reason to justify the combined use of low-quality oils and phenolic extracts. Nevertheless, the results obtained in the present research advise to consider with care the final effects in terms of oxidative and sensory stability and ultimately of shelf-life of the product.

Further investigation is required to assess possible concentration-dependent responses of OPAs supplementation in O/W emulsions either in the oil or the water phase, as well as the effect of changes in the chemical environment. Moreover, the sensory significance of the use of phenolics-rich fractions in food emulsions should also be considered in real applications, in order to address the correct choice of concentrations.

## Figures and Tables

**Table 1:** Parameters and statistics of the Shah fitting models for the decay of hydroperoxides in O/W emulsions, added with OPA, during the first 14 days of accelerated oxidation test with AAPH radical initiator.

	*a*		*b*		*c*		*r*		*x*_0_	*y*_0_	Statistics	
	Value	S.E.	Value	S.E.	Value	S.E.	Value	S.E.	(days)	(mM)	Red χ^2^	Adj. R^2^
Control ^1^	−1.8437	0.65895	0.11807	0.01079	3.34719	0.63524	0.93408	0.01439	9.7	1.03	0.01714	0.99996
O	−0.21181	0.18694	0.04738	0.00383	1.77902	0.18061	0.8843	0.0187	12.4	0.76	0.01199	0.99998
W	0.47324	0.05158	0.02661	0.00133	1.12255	0.0606	0.7466	0.02638	8.6	0.79	0.00464	0.99999

^1^ Control, control emulsion; O, emulsion with OPA added to the oil phase; W, emulsion with OPA added to the water phase.

**Table 2:** Results of the multiple comparisons by Tukey’s HSD test for the data of volatile compounds during accelerated oxidation of O/W emulsions added with OPAs. Mean values ± standard deviation (*n* = 3) ^1^.

Time (d)	Extract	Nonanal	1-Hexanol	Hexanal	Pentanal	1-Pentanol	Propanal	Hex/Pent
	C	A	B	A	A	A	A	B
	O	B	C	B	C	C	C	A
	W	B	A	C	B	B	B	C
0	C	bcd	c	b	d	cd	cd	bcde
	O	de	c	cde	efg	e	defgh	bcd
	W	e	c	f	efg	de	cde	e
2	C	a	c	bc	de	cde	defgh	bcd
	O	cde	c	def	g	e	h	a
	W	e	c	ef	efg	e	def	cde
5	C	ab	c	bcd	def	de	defg	bcd
	O	cde	c	ef	g	e	gh	a
	W	cde	c	ef	fg	e	efgh	bcde
7	C	abc	c	bcd	def	de	fgh	bc
	O	bcde	c	cde	efg	de	efgh	b
	W	bcde	c	ef	efg	de	defgh	de
14	C	f	b	a	a	a	a	f
	O	f	c	ef	c	c	c	f
	W	f	a	ef	b	b	b	F

^1^ Different letters (a–h) on columns mean a significant difference at *p* < 0.05. Capital letters compare emulsions irrespective of oxidation time. Small letters compare emulsions in all sampling times. C, control emulsion; O, emulsion with OPA added to the oil phase; W, emulsion with OPA added to the water phase.

## References

[1] Berton-Carabin C.C., Ropers M.-H., Genot C. (2014). Lipid Oxidation in Oil-in-Water Emulsions: Involvement of the Interfacial Layer. Compr. Rev. Food Sci. Food Saf..

[2] Shahidi F., Zhong Y. (2011). Revisiting the polar paradox theory: A critical overview. J. Agric. Food Chem..

[3] Porter W.L. (1980). Recent Trends in Food Applications of Antioxidants. Autoxidation in Food and Biological Systems.

[4] Decker E.A., McClements D.J., Bourlieu-Lacanal C., Durand E., Figueroa-Espinoza M.C., Lecomte J., Villeneuve P. (2017). Hurdles in Predicting Antioxidant Efficacy in Oil-in-water emulsions. Trends Food Sci. Technol..

[5] Grüneis V., Fruehwirth S., Zehl M., Ortner J., Schamann A., König J., Pignitter M. (2019). Simultaneous Analysis of Epoxidized and Hydroperoxidized Triacylglycerols in Canola Oil and Margarine by LC-MS. J. Agric. Food Chem..

[6] Grüneis V., Pignitter M. (2018). Epoxide Value—A Novel Marker for the Quality Assessment of Food Lipids. J. Agric. Food Chem..

[7] Pignitter M., Zaunschirm M., Lach J., Unterberger L., Kopic A., Keßler C., Kienesberger J., Pischetsrieder M., Eggersdorfer M., Riegger C. (2018). Regioisomeric distribution of 9- and 13-hydroperoxy linoleic acid in vegetable oils during storage and heating. J. Sci. Food Agric..

[8] Paradiso V.M., Pasqualone A., Summo C., Caponio F. (2018). Everything Should Be as Simple as It Can Be. But Not Simpler. Does Food Lipid Oxidation Require an Omics Approach?. Eur. J. Lipid Sci. Technol..

[9] Villeneuve P., Durand E., Decker E.A. (2018). The Need for a New Step in the Study of Lipid Oxidation in Heterophasic Systems. J. Agric. Food Chem..

[10] Laguerre M., Tenon M., Bily A., Birtić S. (2020). Toward a Spatiotemporal Model of Oxidation in Lipid Dispersions: A Hypothesis-Driven Review. Eur. J. Lipid Sci. Technol..

[11] Paradiso V.M., Pasqualone A., Summo C., Caponio F. (2018). An “Omics” Approach for Lipid Oxidation in Foods: The Case of Free Fatty Acids in Bulk Purified Olive Oil. Eur. J. Lipid Sci. Technol..

[12] Gómez-Cortés P., Camiña J.M. (2019). Oxidomics on the omega-3 volatile degradation pattern to determine differences between vegetable and marine oils. Food Res. Int..

[13] Toro-Sierra J., Tolkach A., Kulozik U. (2013). Fractionation of α-Lactalbumin and β-Lactoglobulin from Whey Protein Isolate Using Selective Thermal Aggregation, an Optimized Membrane Separation Procedure and Resolubilization Techniques at Pilot Plant Scale. Food Bioprocess Technol..

[14] International Olive Council (2009). COI/T.20/Doc No 29—Determination of Biophenols in Olive Oils by HPLC.

[15] Reboredo-Rodríguez P., Valli E., Bendini A., Di Lecce G., Simal-Gándara J., Gallina Toschi T. (2016). A widely used spectrophotometric assay to quantify olive oil biophenols according to the health claim (EU Reg. 432/2012). Eur. J. Lipid Sci. Technol..

[16] Singleton V.L., Rossi J.A., Rossi J.A. (1965). Colorimetry of Total Phenolics with Phosphomolybdic-Phosphotungstic Acid Reagents. Am. J. Enol. Vitic..

[17] Re R., Pellegrini N., Proteggente A., Pannala A., Yang M., Rice-Evans C. (1999). Antioxidant activity applying an improved ABTS radical cation decolorization assay. Free Radic. Biol. Med..

[18] Shantha N.C., Decker E.A. (1994). Rapid, sensitive, iron-based spectrophotometric methods for determination of peroxide values of food lipids. J. AOAC Int..

[19] Di Mattia C.D., Paradiso V.M., Andrich L., Giarnetti M., Caponio F., Pittia P. (2014). Effect of Olive Oil Phenolic Compounds and Maltodextrins on the Physical Properties and Oxidative Stability of Olive Oil O/W Emulsions. Food Biophys..

[20] Ricciutelli M., Marconi S., Boarelli M.C., Caprioli G., Sagratini G., Ballini R., Fiorini D. (2017). Olive oil polyphenols: A quantitative method by high-performance liquid-chromatography-diode-array detection for their determination and the assessment of the related health claim. J. Chromatogr. A.

[21] Bellumori M., Cecchi L., Innocenti M., Clodoveo M.L., Corbo F., Mulinacci N. (2019). The EFSA health claim on olive oil polyphenols: Acid hydrolysis validation and total hydroxytyrosol and tyrosol determination in Italian virgin olive oils. Molecules.

[22] Leaver J., Law A.J.R., Brechany E.Y., McCrae C.H. (1999). Chemical changes in β-lactoglobulin structure during ageing of protein-stabilized emulsions. Int. J. Food Sci. Technol..

[23] Leaver J., Law A.J.R., Brechany E.Y. (1999). Covalent modification of emulsified β-casein resulting from lipid peroxidation. J. Colloid Interface Sci..

[24] Kamal-Eldin A., Kamal-Eldin A. (2003). Lipid Oxidation Pathways.

[25] Horn A.F., Nielsen N.S., Jensen L.S., Horsewell A., Jacobsen C. (2012). The choice of homogenisation equipment affects lipid oxidation in emulsions. Food Chem..

[26] Shah B.K. (1961). A Simple Method of Fitting the Regression Curve y = α+ δx + βρ x. Biometrics.

[27] Aragao G.M.F., Corradini M.G., Peleg M. (2008). A phenomenological model of the peroxide value’s rise and fall during lipid oxidation. JAOCS, J. Am. Oil Chem. Soc..

[28] Vlachos N., Skopelitis Y., Psaroudaki M., Konstantinidou V., Chatzilazarou A., Tegou E. (2006). Applications of Fourier transform-infrared spectroscopy to edible oils. Anal. Chim. Acta.

[29] Hayati I.N., Man Y.B.C., Tan C.P., Aini I.N. (2005). Monitoring peroxide value in oxidized emulsions by Fourier transform infrared spectroscopy. Eur. J. Lipid Sci. Technol..

[30] Daoud S., Bou-maroun E., Dujourdy L., Waschatko G., Billecke N., Cayot P. (2019). Fast and direct analysis of oxidation levels of oil-in-water emulsions using ATR-FTIR. Food Chem..

[31] Forss D.A. (1973). Odor and flavor compounds from lipids. Progress in the Chemistry of Fats and Other Lipids.

[32] Schaich K.M. (2005). Lipid Oxidation: Theoretical Aspects. Bailey’s Industrial Oil and Fat Products.

[33] Paradiso V.M., Di Mattia C., Giarnetti M., Chiarini M., Andrich L., Caponio F. (2016). Antioxidant Behavior of Olive Phenolics in Oil-in-Water Emulsions. J. Agric. Food Chem..

[34] Meynier A., Rampon V., Dalgalarrondo M., Genot C. (2004). Hexanal and t-2-hexenal form covalent bonds with whey proteins and sodium caseinate in aqueous solution. Int. Dairy J..

[35] Benjamin O., Leus M., Everett D.W. (2011). Static headspace analysis of volatile compounds released from β-lactoglobulin-stabilized emulsions determined by the phase ratio variation method. Food Res. Int..

[36] Tavel L., Andriot I., Moreau C., Guichard E. (2008). Interactions between β-lactoglobulin and aroma compounds: Different binding behaviors as a function of ligand structure. J. Agric. Food Chem..

[37] Van Ruth S.M., King C., Delarue M., Giannouli P. (2002). Release of volatile compounds from emulsions: Influence of β-lactoglobulin and pH. Ital. J. Food Sci..

[38] Guichard E., Langourieux S. (2000). Interactions between β-lactoglobulin and flavour compounds. Food Chem..

[39] Nuchi C.D., Hernandez P., McClements D.J., Decker E.A. (2002). Ability of Lipid Hydroperoxides To Partition into Surfactant Micelles and Alter Lipid Oxidation Rates in Emulsions. J. Agric. Food Chem..

[40] Kharasch E.D., Novak R.F. (1985). Mitoxantrone and ametantrone inhibit hydroperoxide-dependent initiation and propagation reactions in fatty acid peroxidation. J. Biol. Chem..

[41] Carrasco-Pancorbo A., Cerretani L., Bendini A., Segura-Carretero A., Del Carlo M., Gallina-Toschi T., Lercker G., Compagnone D., Fernández-Gutiérrez A. (2005). Evaluation of the antioxidant capacity of individual phenolic compounds in virgin olive oil. J. Agric. Food Chem..

[42] Chimi H., Cillard J., Cillard P., Rahmani M. (1991). Peroxyl and hydroxyl radical scavenging activity of some natural phenolic antioxidants. J. Am. Oil Chem. Soc..

[43] Paiva-Martins F., Gordon M.H. (2002). Effects of pH and ferric ions on the antioxidant activity of olive polyphenols in oil-in-water emulsions. J. Am. Oil Chem. Soc..

[44] Difonzo G., Pasqualone A., Silletti R., Cosmai L., Summo C., Paradiso V.M., Caponio F. (2018). Use of olive leaf extract to reduce lipid oxidation of baked snacks. Food Res. Int..

